# Editorial: Role of serotonergic system in pathology of major depressive disorders

**DOI:** 10.3389/fpsyt.2022.974388

**Published:** 2022-07-15

**Authors:** Gohar Fakhfouri, Reza Rahimian

**Affiliations:** ^1^Department of Psychiatry, Douglas Hospital, McGill University, Montreal, QC, Canada; ^2^McGill Group for Suicide Studies, Douglas Mental Health University Institute, Verdun, QC, Canada

**Keywords:** major depressive disorder (MDD), serotonin, biomarker, serotonin transporters, 5-HT receptors

The monoamine serotonin (5-HT) is involved in a breadth of physiological processes from modulating metabolism to mood and cognition. Several mechanisms control the synaptic bioavailability of 5-HT and its postsynaptic responses. These are exemplified by different transporters and the occurrence of seven distinct receptors (5-HT1-7), some bearing subtypes. 5-HT receptors differ in terms of localization and downstream signaling, adding to the complexity of the 5-HT system. 5-HT has been strongly linked to major depressive disorder (MDD) and is the subject of numerous experimental and human studies. The role of the 5-HT transporters has been established in MDD, while the majority of the known 5-HT receptors have also been implicated in MDD or depressive-like behavior.

The articles published in this Research Topic contribute to advancing our knowledge on the complex role of serotonergic system, its receptors and metabolizing enzymes in MDD and interrogate the possibility of novel biomarkers as well as therapeutic targets for MDD.

In their review article, Vahid-Ansari and Albert discuss the evidence that 5-HT governs the wiring of the developing brain, both by modulating 5-HT innervation and by influencing synaptic organization within corticolimbic structures. They summarize role of some key 5-HT receptors along with their possible downstream signaling underlying the axonal growth and axonal guidance. In addition, given that accumulating evidence suggests that deficiencies in 5-HT innervation associated with aberrant development, chronic stress or brain insult may lead to depression, and that the 5-HT system is capable of regenerating lost projections, they postulate that by amplifying the rewiring processes of the 5-HT system using SSRIs and selective 5-HT agonists, it is conceivable to achieve more rapid and effective treatments for stress- or injury-induced depression or cognitive impairment.

The etiology of MDD and the response rate to its medications are believed to be governed by a combination of environmental, psychological and genetic factors (1). Identifying universal or ethnic genetic polymorphisms that predispose to MDD or suicidal behavior as well as those predicting treatment response to distinct drug classes could provide practitioners with valuable diagnostic and therapeutic biomarkers. The meta-analysis by Yang et al. found the association of two functional single nucleotide polymorphisms in *HTR1B* with the risk of MDD and suicidal behavior. The 5-HT1B receptor, encoded by the gene, is a G_i_-coupled receptor widely distributed in the brain with mainly presynaptic cellular localization that acts as auto- and hetero-receptor, thereby playing an essential role in modulating the release of 5-HT and other neurotransmitters (2).

Monoamino Oxidase A (MAOA), a key enzyme in metabolism of 5-HT and other monoamine neurotransmitters and the target of MAO inhibitor class of antidepressants, has been extensively associated with suicidal behavior. In an attempt to identify genetic and epigenetic correlates of violent suicide, Ludwig et al. uncovered an association between violent suicide attempts and a single nucleotide polymorphism within *MAOA* gene as well as hypomethylation of the MAOA gene Exon I promoter region in female affective disorder patients.

The imaging study led by de Cates et al. provided further support for a pro-cognitive effect of 5-HT4 receptor agonism in humans. Indeed, 5-HT4 receptor agonists have proved promising in both animal models of depression and cognitive deficit. New generation of 5-HT4 receptor agonists elicit rapid onset of action and low incidence of side effects (3).

Several lines of evidence indicate that neuroinflammation plays a pivotal role in MDD pathogenesis. Levels of different cytokines is altered in a subset of depressed patients. Interestingly, many neurological disorders are characterized by a high incidence of depression along with marked neuroinflammation. Moreover, inflammatory cytokines are increased in the blood and CSF of a subset of depressed patients. Accordingly, antidepressants such as SSRIs can modulate glial activity and regulate the expression of several cytokines and chemokines (4). The interleukin-8 (IL-8) has been reported to play a role in depression, which might be modulated by the selective serotonin reuptake inhibitors (SSRIs). The research by Zhu et al. found that MDD was associated with a decline in serum IL-8 levels and that surprisingly treatment with SSRI failed to restore the blood levels of IL-8.

An intriguing research study by Kesic et al. revealed that higher constitutional 5HT activity is associated with higher sensitivity to anxiolytic effects of fluoxetine. In addition, their findings highlighted the role of endogenous 5HT tone as an important factor influencing behavioral response to antidepressants. Finally, they showed plasma 5HT level as a predictor of individual response to SSRI treatment, indicating its potential value as an early biomarker of therapeutic response in clinical settings. On the other hand, 5-HT concentration in saliva, which appears to reflect peripheral more than central 5-HT content was shown by Karbownik and Hicks to be negatively associated with current mood in healthy individuals.

## Author contributions

All authors listed have made a substantial, direct, and intellectual contribution to the work and approved it for publication.

## Funding

GF and RR are FRQ-S fellows.

## Conflict of interest

The authors declare that the research was conducted in the absence of any commercial or financial relationships that could be construed as a potential conflict of interest.

## Publisher's note

All claims expressed in this article are solely those of the authors and do not necessarily represent those of their affiliated organizations, or those of the publisher, the editors and the reviewers. Any product that may be evaluated in this article, or claim that may be made by its manufacturer, is not guaranteed or endorsed by the publisher.
